# Effect of Scanning Strategy on Thermal Stresses and Strains during Electron Beam Melting of Inconel 625: Experiment and Simulation

**DOI:** 10.3390/ma16010443

**Published:** 2023-01-03

**Authors:** Xiaoyu Zhao, Yuan Wei, Rami Mansour, Sasan Dadbakhsh, Amir Rashid

**Affiliations:** 1 Department of Production Engineering, KTH Royal Institute of Technology, Brinellvägen 68, 11428 Stockholm, Sweden; 2 Frontiers Science Center for Flexible Electronics, Xi’an Institute of Flexible Electronics (IFE), Xi’an Institute of Biomedical Materials & Engineering, Northwestern Polytechnical University, Xi’an 710072, China; 3 Department of Engineering Mechanics, KTH Royal Institute of Technology, Teknikringen 8D, 10044 Stockholm, Sweden

**Keywords:** thermal distortion, scanning strategy, electron beam melting (EBM), additive manufacturing, simulation

## Abstract

This paper develops a hybrid experimental/simulation method for the first time to assess the thermal stresses generated during electron beam melting (EBM) at high temperatures. The bending and rupture of trusses supporting Inconel 625 alloy panels at ~1050 °C are experimentally measured for various scanning strategies. The generated thermal stresses and strains are thereafter simulated using the Finite-Element Method (FEM). It is shown that the thermal stresses on the trusses may reach the material UTS without causing failure. Failure is only reached after the part experiences a certain magnitude of plastic strain (~0.33 ± 0.01 here). As the most influential factor, the plastic strain increases with the scanning length. In addition, it is shown that continuous scanning is necessary since the interrupted chessboard strategy induces cracking at the overlapping regions. Therefore, the associated thermal deformation is to be minimized using a proper layer rotation according to the part length. Although this is similar to the literature reported for selective laser melting (SLM), the effect of scanning pattern is found to differ, as no significant difference in thermal stresses/strains is observed between bidirectional and unidirectional patterns from EBM.

## 1. Introduction

Additive manufacturing (AM) is a layer-based process to selectively melt/fuse the material based on the sliced CAD model. Amongst several, the powder bed fusion (PBF) is the most mature and widely used metal AM technique. Within PBF processes, selective laser melting (SLM) and electron beam melting (EBM) are the most common. Both processes apply a high-energy source, such as a laser or electron beam, to melt the metallic powder, layer by layer, until solid parts are built [1]. This high-energy source can create a melt pool temperature as high as 2500–3500 °C in order to fully melt the metal powder [2,3,4,5]. Subsequently, the beam continuously moves to other places, while the molten materials are rapidly solidified at a rate of at least 103–104 °C/s [2,3,4,6]. This can lead to an uneven surface/volume temperature and a large temperature gradient, consequently, large thermal and residual stresses.

The induced stresses can be either in tensile or compression modes. For a single layer, during heating, the material is melted by a high-energy source. The melting region tends to expand and induce compressive strains to the surrounding materials. Afterward, during cooling, the melt pool is solidified and shrunk towards the center. Therefore, tensile stress is generated within the solidified pool as well as the surrounding material. Thereafter, due to the subsequent expansion and contraction of the melt pool, a single layer easily bends if it is not anchored by a substrate. The situation is even more complex when adding successive layers. This causes solid material remelting and fusion to previous layers, restricted by the surrounding material. This further causes a reverse compressive deformation, which may deform the material and/or generate residual stresses [7,8,9].

The deformation is observed in both EBM and SLM. However, there is a significant difference in the magnitude of such stresses between these processes. This is due to the most notable difference between them, i.e., the high EBM processing temperature. For the EBM process, before adding the powder, the starting baseplate is directly preheated to the intended temperature. Only after reaching the intended temperature does the EBM printing starts. Therefore, EBM produces components under an extremely high processing temperature, in the range of 730–1100 °C for different materials under a vacuum [10,11,12]. SLM is conducted at a much lower temperature which could be around 100–400 °C [13,14]. Therefore, in SLM, the low processing temperature [15,16] and the steep temperature gradient [17] can lead to larger residual stresses. In comparison, the high-temperature working environment of the EBM process can be thought of as an in-situ stress-relief heat treatment, and hence resulting in residual-stress-free products [18,19].

In SLM, the thermal stresses and the resultant residual stresses (those remaining in the part after cooling down) are extensively studied and analyzed [9,20,21]. In contrast, these aspects are rather neglected for EBM due to the active recovery phenomena at high temperatures that leave almost no residual stress when the part is cooled to room temperature [22]. Despite this, EBM can still generate thermal stresses [23] from the applied temperature gradient. Once these stresses are large enough, they can even bend the baseplate and/or cause delamination between layers. This can reduce the part’s geometrical quality or even cause collisions between the part and the raking system. Influential factors on thermal stress or delamination failure have been explored in several studies. Studies by Kahnert et al. [24] and Zäh et al. [25,26] analyzed the delamination failure of the tool steels H11, H13 and stainless steel 316L in EBM. It is shown that the delamination failure is attributed to the lack of adhesion between the layers. Accordingly, higher energy input to fully melt the powder can increase the adhesion between the layers and restrict the deformation of the top layer [24,25,26]. Cheng and Chou [8,27] have analyzed the need of overhanging surfaces for the support of EBM of Inconel 718 (IN718). They have found that increasing the electron beam energy density can increase the deformation of the overhanging features due to the higher local temperatures. This was while the simulated stresses remained rather similar to that of the parts made by lower energy densities. Moreover, although a solid substrate could anchor/suppress the deformation, it consequently increased the thermal stresses [8,27]. Similarly, Ameen et al. [28] reported that the deformation increased with increasing the energy input (e.g., higher beam current and lower beam speed, etc.) for EBM-made Ti-6Al-4V overhanging features [28].

In addition to poor process parameters, the oversized part dimensions can enlarge the thermal stresses and the part distortions [15,29,30]. Deformation of the EBM parts can be high enough to potentially distort the baseplate as well. Prabhakar et al. [23] conducted a simulation work to investigate the formation of the wrapping of the baseplate after the EBM of IN718 material. They concluded that the warping could happen during the first few layers of EBM, and it can increase during cooling due to the cooling rate differences between the baseplate and the solid components [23].

**Figure 1:**
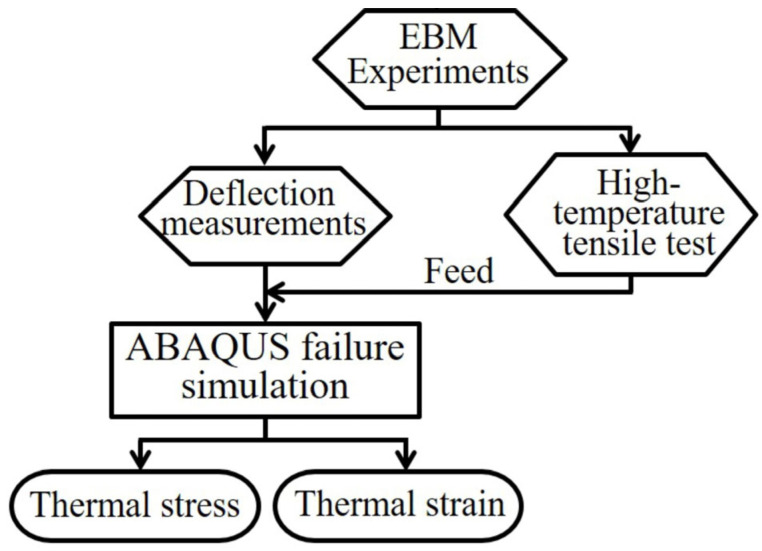
A flowchart of the experimental and simulation works applied in this research.

Despite the above-mentioned literature, the thermal stresses and their correspondence to the applied scanning strategy have remained unexplored for EBM (although, in comparison, they are rather well recognized in SLM [9,20,21]). Therefore, a systematic study to analyze the influence of the EBM scanning strategy on the generation of thermal stresses and the corresponding deformations is necessary. This is particularly interesting for the case of Inconel 625 (IN625), which is still in the explorative phase as a promising material for AM. Accordingly, this work aims at exploring this unknown area. The procedure applied in this work, to analyze the thermal deformations and to estimate the thermal stresses during EBM of IN625 resulting from various scanning strategies, is shown in the flowchart in Figure 1. After EBM experiments, deflection measurements are performed to assess the influence of scanning lengths, bidirectional scanning, layer rotation and chessboard scanning, on the thermal deformation. A hypothesis is set according to the observations from the experiments. High-temperature tensile testing is also performed to obtain the needed mechanical property of IN625. Due to extreme difficulties to create a layer-wise simulation for failure in EBM; the experimental deformation and material properties at high temperatures are used as an input to ABAQUS for creating a simulation to visualize the thermal stresses/strains during EBM. The outputs from ABAQUS simulations are later compared with the experimental result to validate the correctness of the hypothesis.

## 2. Experiments and Methods

### 2.1. EBM Processing

**Figure 2:**
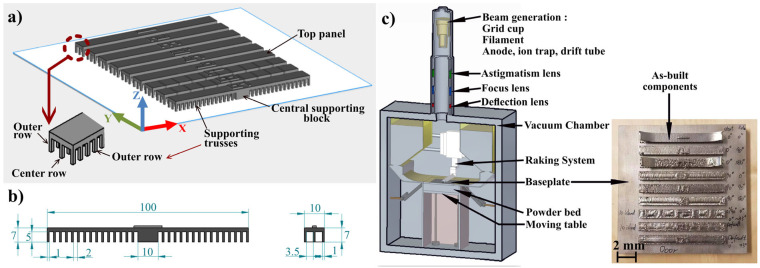
(**a**) Printing layout, (**b**) size and (**c**) the Arcam EBM system used for this study and visual condition of the deflection testing parts after EBM.

The commercial Osprey^®^ Alloy 625, provided by Sandvik (Sandviken, Sweden) was used in this study. This powder was gas atomized in a spherical shape with an average equivalent size of 75 µm and 95% ranging from 45 to 105 µm [31]. EBM Arcam A2X machine (GE Additive, Mölnlycke, Sweden) was used to produce the testing specimens using a stainless steel baseplate. The baseplate was 170 × 170 mm^2^ in cross-section and 10 mm in thickness. The testing specimens were directly built on top of the baseplate as shown in Figure 2.

As seen, these specimens consisted of three regions: (i) the central supporting block with a size of 10 × 10 × 5 mm^3^, (ii) three rows of symmetrically distributed supporting trusses with a size of 1 × 1 × 5 mm^3^ and a spacing of 2 mm between adjacent ones, and (iii) the top panel with a size of 10 × 100 × 2 mm^3^.

For EBM, the general processing parameters used to achieve a dense product were used to produce these test samples, as studied in our previous research [31]. Accordingly, during EBM, the baseplate was preheated to a temperature of 1025 °C before the layerwise printing. This was maintained by measuring the temperature underneath the baseplate. After heating the naked baseplate to the desired temperature, the powder was dispersed for layer-wise building. During the building after each layer of powder dispersion, heating was applied both before and after the melting stage to maintain the high temperature. Moreover, the automation mode in EBM control software was activated for manufacturing the samples. The filament voltage was 60 kV and the powder layer thickness was 0.075 mm. All the samples were printed with only hatching, with a line space of 0.042 mm, a line order of 1, and both bidirectional and unidirectional scanning patterns. The samples yielding extreme bending were printed only once since they can destroy the raking system. All other samples were printed twice. Different processing parameters including start scanning angle, rotation angle per layer and chessboard printing were practiced, as shown in Table 1.

**Figure 3:**
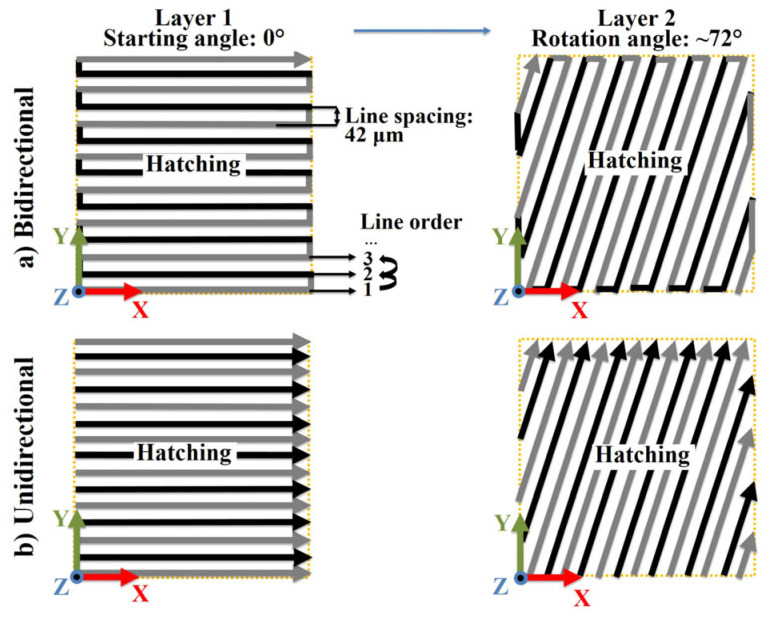
Schematic of the default scanning strategy with a starting angle of 0° and a rotation angle of ~72°: with (**a**) bidirectional and (**b**) unidirectional scanning.

**Figure 4:**
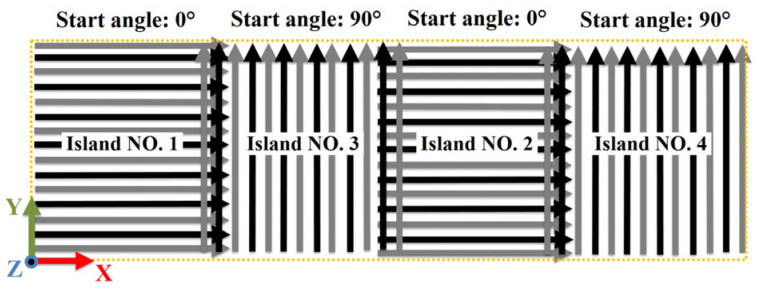
Schematic of the unidirectional chessboard printing. As shown, the chessboard printing was achieved by dividing the entire slice into several (10 for this work) islands, where each island was melted one after another.

In order to elaborate on the idea about the altered parameters, the ‘snake function’ and the ‘chessboard printing’ are schematically shown in Figure 3 and Figure 4. The arrows show the beam movement directions. As seen, Figure 3a shows the default scanning strategies with the ‘snake’ function true. By using this function, the electron beam moved in a bidirectional pattern. In comparison, the beam only moves unidirectionally without this ‘snake’ function. Furthermore, as shown in Figure 4, the chessboard printing was achieved by dividing the entire slice into several islands (10 islands in this work) having an overlapping region of 0.8 mm. The islands with the same scanning angle (0° or 90°) were melted one after another. Accordingly, the islands were melted following the order from NO. 1 to NO. 4 as shown in Figure 4. Moreover, Table 2 summarizes the scanning angle, length and time for different parts.

### 2.2. Measurement and Testing

**Figure 5:**
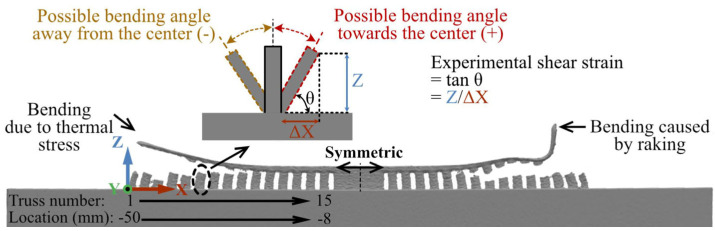
Three-dimensional scanned unidirectional S0R180 part, showing the typical bending of the supporting trusses.

As shown in Figure 2, there were two outer rows and one middle row of the supporting trusses. Due to the limitation of the 3D scanning, the trusses aligned in the middle row could not be scanned. Accordingly, the samples were sliced between the rows (Figure 2a), using an electron discharge machining (EDM) system (Sodick VL600Q, Kanagawa, Japan). Afterward, the sliced samples were 3D scanned using GOM ATOS Core 200 (GOM mbH, Braunschweig, Germany) with a resolution of 80 µm. The scanned and collected data were analyzed using the software GOM Inspect V7.5 SR2. From the scanned data, the size of two supporting trusses from each side of the central block was measured (as they were almost un-bended). Accordingly, the actual truss sizes were used for the analysis. Nevertheless, the supporting trusses showed two types of deformations, i.e., bend and rupture from the top panel (Figure 5). The bending could either occur in the negative (away from the central supporting block) or positive (towards the central supporting block) directions. The angle ‘θ’ was measured between the baseplate and the truss from the scanned parts. Afterward, the shear strain was derived experimentally as tanθ or the ratio between the actual height of the truss and the displacement along the x-axis (see tanθ = Z/∆X in Figure 5). It should be noted that since the trusses were bent in a curved manner (they were not straight), the maximum bending angle from the top part of the trusses was measured to calculate the maximum shear strain.

Moreover, the bending angles on the trusses were measured from the same scan but at three different locations within the x-z plane: the top left, the top middle and the top right points of the trusses. In addition, for the symmetrically bent parts, the trusses from both sides were analyzed to obtain the average and the deviation of the bending angles. In comparison, the unsymmetrically bent parts were analyzed from both outer rows (except the middle row) of the trusses.

After the 3D scanning, the top panels were cut out using the same EDM for density analysis, in order to verify the performance of different scanning strategies. The densities were measured using Archimedes’ method according to standard ASTM B311-17 [32]. The weighing liquid was isopropanol 99.5% with a density of 0.7842 g/cm^3^ at 21 °C. Thereafter, these samples were ground and polished according to standard metallographic preparation procedures to a 1 μm finish. The defect analyses were performed on these polished samples using the Scanning Electron Microscope (SEM), Phenom ProX.

**Figure 6:**
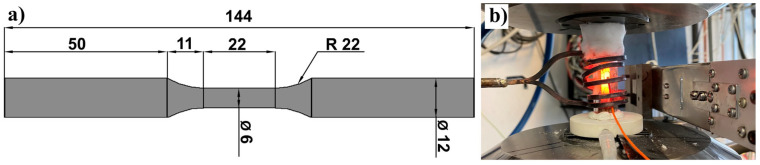
(**a**) The specimen drawing for high temperature tensile testing (unit: millimeter), and (**b**) the high temperature tensile testing set-up.

In order to examine the material properties at high temperatures in the EBM operations, two blocks with a size of 14 × 114 × 28 mm^3^ were printed. They were horizontally printed to increase the similarity to the printed panels. These samples were diagonally placed on the baseplate and produced with the default settings (part S0R72 in Table 1 and Table 2). After EBM, these blocks were deeply machined (a 3.5 mm machining depth in the reduced section) to the size shown in Figure 6a. After machining, the specimens were tensioned on MTS3 (MTS, Huntsville, AL, USA) at 1050 °C, achieved with induction heating (Figure 6b), and a shielding gas of Argon with a purity of 99.99%. For these tests, the pulling rate was 1 mm/min.

### 2.3. Simulation and Modeling

**Figure 7:**
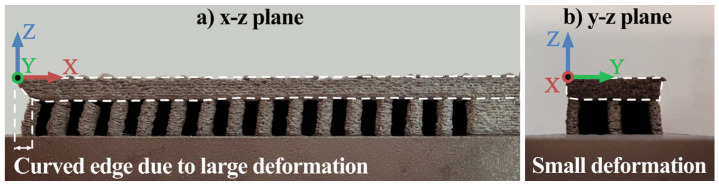
The deformation of the top panel for bidirectional S0R90 part, in (**a**) *x*-axis and (**b**) *y*-axis. As seen, large distortions could be observed along *x*-axis while there is no significant distortion along the *y*-axis.

Within the existing research works, many simulations for the SLM process were conducted layer-wise. However, this was not possible in our case due to several reasons: (i) no commercial software was found to simulate failure with layer-wise algorithms, (ii) the differences in the melt pool from laser vs. electron beam are not exactly clear, as the current commercial software is only used for SLM simulations, (iii) there are a lack of material data for high-temperature simulations, as applied in EBM. Therefore, herein we aimed at developing a physical simulation, based on observed experimental deformations and failures. To do so, after experimental measurement of the bending for the supporting trusses and top panel, a 2D explicit Finite-Element (FE) model using ABAQUS v6.14-1 CAE was developed for estimating thermal stresses on the supporting trusses (Figure 7). In the printed components, a clear bending was observed along the *x*-axis, while comparatively, negligible bending occurred along the width (*y*-axis) particularly for the middle trusses, as shown in Figure 7. Accordingly, for the current study, only a 2D simulation was applied to estimate the stresses on each truss in order to simplify the analysis.

#### 2.3.1. Simulation Assumptions

According to the design of deflection testing specimens, the supporting trusses had a very small area of 1 × 1 mm^2^ (Figure 2). Consequently, the deformation of trusses before printing the top panel was assumed to be negligible. During the printing of the top panel, the application of the high-energy electron beam continuously caused solid/liquid phase changes. Therefore, volume changes continuously imposed a thermal load on the panel. These volume changes in the top panel could deform and even rupture the supporting trusses. To simplify the simulation analysis, the deformation and rupture of the trusses were assumed to be caused by the lateral shrinkage of the solid top panel.

**Figure 8:**
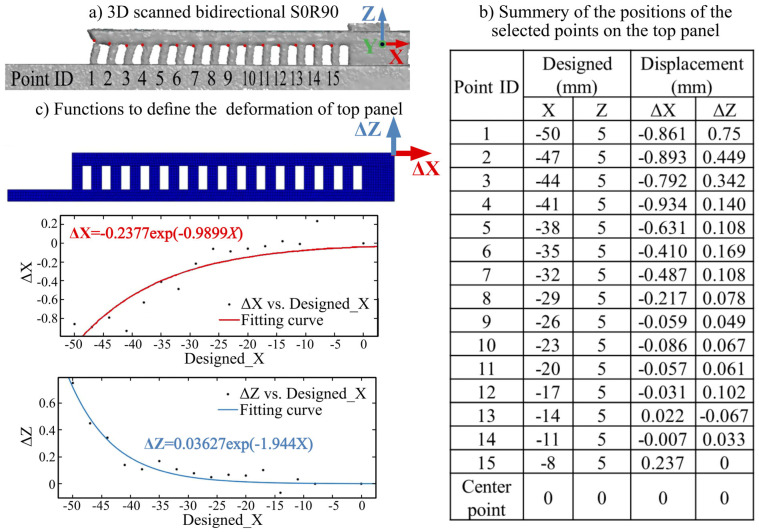
A typical example, experimentally realizing the top panel deformation from bidirectional S0R90 part: (**a**) 3D scanned result of the selected points. (**b**) Summary of the designed positions, the displacement of the selected points along *x*- and *z*-axis. (**c**) Displacement functions realized from (**b**), ∆X(X) and ∆Z(X), applied to the FE-model to describe the final deformed model.

This simulation used the deformation/curvature of the top panel as inputs to analyze the stresses and strains on the trusses. The curvature of the top panel was experimentally measured and formulated as two functions along the *x*- and *z*-axis to define the post-failure bending of the panel. These panel bending functions are thereafter applied in the simulation. Figure 8 shows the procedure for defining the curvature on the top panel for the bidirectional S0R90 part. After 3D scanning (Figure 8a), the actual location of at least 15 connection points (between the top panel and trusses) is summarized and compared with the designed location (Figure 8b). The functions were thereafter formulated from the designed x-position vs. ∆X and the designed x-position vs. ∆Z, as shown in Figure 8. The stresses on each truss could, therefore, be simulated in ABAQUS.

To make the simulation consistent with the experimental observations, the stresses were simulated according to the top panel deformation in Figure 8. The deformation of the top panel ends when it reaches the experimentally defined curvature. In addition, it is interesting to note that a few trusses were ruptured below the top panel near the middle of the truss (less than 10% of the trusses). However, since this was a minority, their influence on the top panel deformation was not considered in this study.

It should be mentioned that the temperature during EBM processing was measured below the baseplate as 1025 °C. However, the actual printing was performed above the baseplate. Naturally, this should be slightly higher than the bottom temperature. Therefore, a temperature of 1050 °C was assumed as the actual processing temperature. Accordingly, the material properties were tested (as summarized in Table 3) and the thermal stresses and strains were simulated at 1050 °C. Furthermore, due to the limited height of the supporting trusses, the temperature difference along the building direction on the deflection testing samples was considered negligible.

Due to the high processing temperature of 1050 °C, it was assumed that there was no residual stress remaining in the material (due to the material recovery mechanisms active at high temperatures). After EBM, the deflection samples were also slowly cooled to room temperature with a rate of ~0.07–0.08 °C/s, taking over 3–4 h in a vacuum as the default condition of the process [33]. Accordingly, due to this very slow cooling and no residual stresses from processing at high temperatures, the further bending of trusses during cooling was assumed to be negligible.

#### 2.3.2. Simulation Settings

In the FE analysis, the initiation and propagation of interface delamination is simulated using cohesive elements [34]. A thin cohesive zone with a thickness of 0.1 mm was aligned at the interfaces of the supporting trusses and the top panel as a sacrificing layer. This was automatically deleted when the rupture happened. The 4-node bilinear plane strain quadrilateral mesh (ABAQUS CPE4R mesh) was applied to the simulation models. This mesh was generated according to the part size, with four Finite-Elements along the width of each truss. Therefore, the exact mesh size among the samples might be slightly different. A mesh convergence study was conducted which assured that the chosen mesh size was appropriate in terms of stress evaluation.

In the failure simulation, damage initiation occurs when the accumulated equivalent plastic strain during the deformation process reaches a critical value. That is, the damage initiation criteria are given by

(1) $$\int d{\bar{\varepsilon }}^{pl}={\bar{\varepsilon }}_{cr}^{pl},$$

where $d{\bar{\varepsilon }}^{pl}$ is the incremental equivalent plastic strain and ${\bar{\varepsilon }}_{cr}^{pl}$ is the critical value for which damage is initiated. The critical value was determined based on the studied samples by matching the number of failed trusses from the experiment. The criteria were implemented based on ABAQUS built-in model [35,36,37].

After initiation, a linear damage evolution law of $dD=d{\bar{u}}^{pl}/{\bar{u}}_{f}^{pl}$ was applied to simulate the propagation of the interface delamination until final failure. Here, ‘*D*’ is changing from ‘0′ at damage initiation to ‘1’ at final failure. Additionally, ${\bar{u}}^{pl}$ is the equivalent plastic displacement defined as the fracture work conjugate of the yield stress after the onset of damage [38,39], ${\bar{u}}^{pl}=0$ at damage initiation and ${\bar{u}}^{pl}={\bar{u}}_{f}^{pl}$ at final failure. The equivalent plastic displacement at failure is given by

(2) $${\bar{u}}_{f}^{pl}=L\;\left({\bar{\varepsilon }}_{f}^{pl}-{\bar{\varepsilon }}_{0}^{pl}\right),$$

where ${\bar{\varepsilon }}_{f}^{pl}$ and ${\bar{\varepsilon }}_{0}^{pl}$ denote the plastic strain at failure and equivalent plastic strain at the onset of damage, respectively. These are obtained experimentally from high-temperature tensile testing. The characteristic length *L* represents the cohesive element size. It is noted that *L* varied slightly among the samples according to the actual width of the truss.

## 3. Results

### 3.1. High-Temperature Tensile Properties

**Figure 9:**
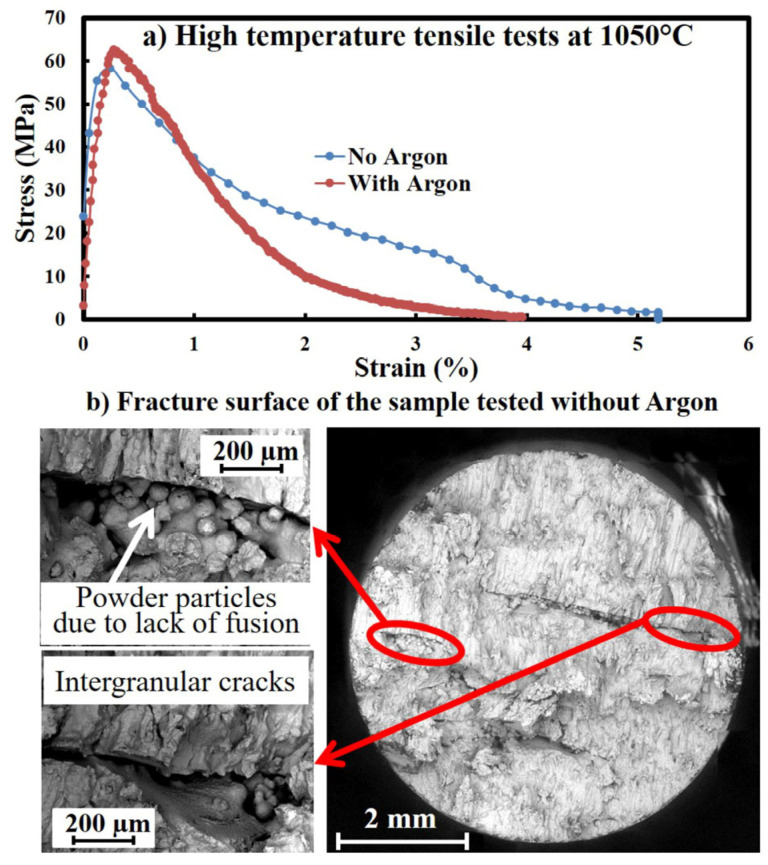
(**a**) The tensile property of the as-built EBM IN625 samples at 1050 °C. (**b**) Fracture surface of the samples tested without shielding gas. Large lack of fusion defects and intergranular cracks can be observed in the inserts.

Figure 9 shows the tensile properties and the fracture surface of the machined samples at 1050 °C. As seen, the samples can only reach a UTS of about 58 MPa and an elongation of about 5%. This is approximately 10% of both the UTS and elongation at room temperature [31]. The drastic drop in the UTS is natural at this high temperature [40]. It can mainly be attributed to the severe embrittlement of this alloy, after AM, above 870 °C. This is reported for SLM-made IN625 parts at 871 °C and can be attributed to the distribution of the M6C precipitates along grain boundaries. Accordingly, the carbide precipitation weakens the grain boundaries by promoting intergranular cracks after the material yield [41,42,43]. This has also occurred in our work, as large intergranular cracks rapidly formed and developed after the yield point (Figure 9b). Accordingly, these cracks dropped the bearable strength and rapidly deteriorated the plastic deformation of the material. Since this has suppressed the natural development of plastic deformation, the tensile stress-strain curve seems neither plastic nor brittle (Figure 9a). In addition, the EBM defects (such as the lack of fusion with a multitude of non-molten powder particles) can also contribute to the lower strength, cracking and premature failure of the parts (Figure 9b). It is interesting to reiterate that the tension was conducted on the horizontal parts at 1050 °C to be in line with the manufactured panels, although the mechanical strength might be varied for the vertically made samples at this temperature.

### 3.2. Truss Size and Defect Analysis

Table 4 summarizes the truss size of the as-built samples. As can be seen, typically the truss widths (*x*-axis) and thicknesses (*y*-axis) were larger than the designed size of 1 mm. This is possibly explained by the use of automatic mode, which is originally designed to improve the production rate by merging the small scanning lines along the same direction to an entire line [44]. In automatic mode, the beam jumps at an extremely high speed between scanning lines, which can lead to inaccurate movement in the direction of scanning. This has caused a small position shift as well as truss size variations. Moreover, the actual height (*z*-axis) was less than the designed value of 5 mm (Table 4). This could be attributed to the down penetration of the top panel, warping of the baseplate as well as the manual insertion of the baseplate onto the powder cake, influencing the initial leveling in EBM.

**Figure 10:**
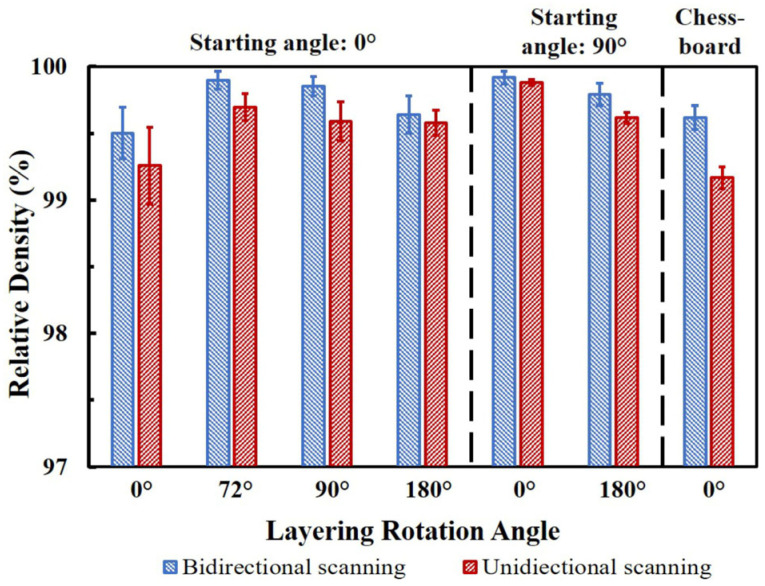
Density of parts made using different scanning strategies. Note: the scale bar starts from 97%, and all the samples showed over 99% density.

**Figure 11:**
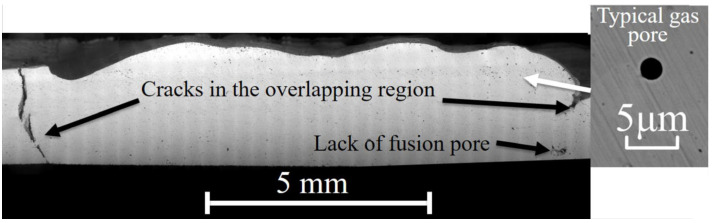
The defects formed at the overlapping region of the 10I:0/90R0 part.

Figure 10 demonstrates the density of the as-built parts. In general, all the parts were over 99% density with some lack of fusion and gas pores. However, the density was slightly changed according to the starting angle, layer rotation angle and the bidirectional/unidirectional scanning pattern. For example, the samples with bidirectional melting always showed a higher density. This could be due to the pause between scanning lines in a unidirectional pattern that can cause a slight decline in the temperature and consolidation. This can be confirmed by the longer scanning time for unidirectional scanning, as presented in Table 2. Furthermore, for the long scanning parts, the commercially recommended setting with a 72° rotation angle (part S0R72) led to the highest density. This was, however, still comparable to the part with 90° rotation (part S0R90), as seemingly any rotation between the layers can efficiently consolidate the defects between the additive layers. In fact, short-Y scanning lines (S90R0 and S90R180) can lead to the highest densities, as they can logically produce higher temperatures on the working layer. However, the density of the parts was reduced after applying the chessboard strategy. This can be associated with the large cracks formed at the overlapping regions between the islands, as shown in Figure 11. These cracks are the result of regional melting and solidification/shrinkage in possibly opposite directions.

### 3.3. Deflection and Thermal Stress Analysis Truss Size and Defect Analysis

In the current experiments, there were two distinct types of deformation: (i) lateral bending of trusses due to the shrinkage of the panel and (ii) the separation/rupture of the trusses due to the extreme bending of the top panel. According to these bending, the thermal stresses/strains during EBM are simulated with the aid of ABAQUS, as explained below.

#### 3.3.1. Long Scanning Parts

**Figure 12:**
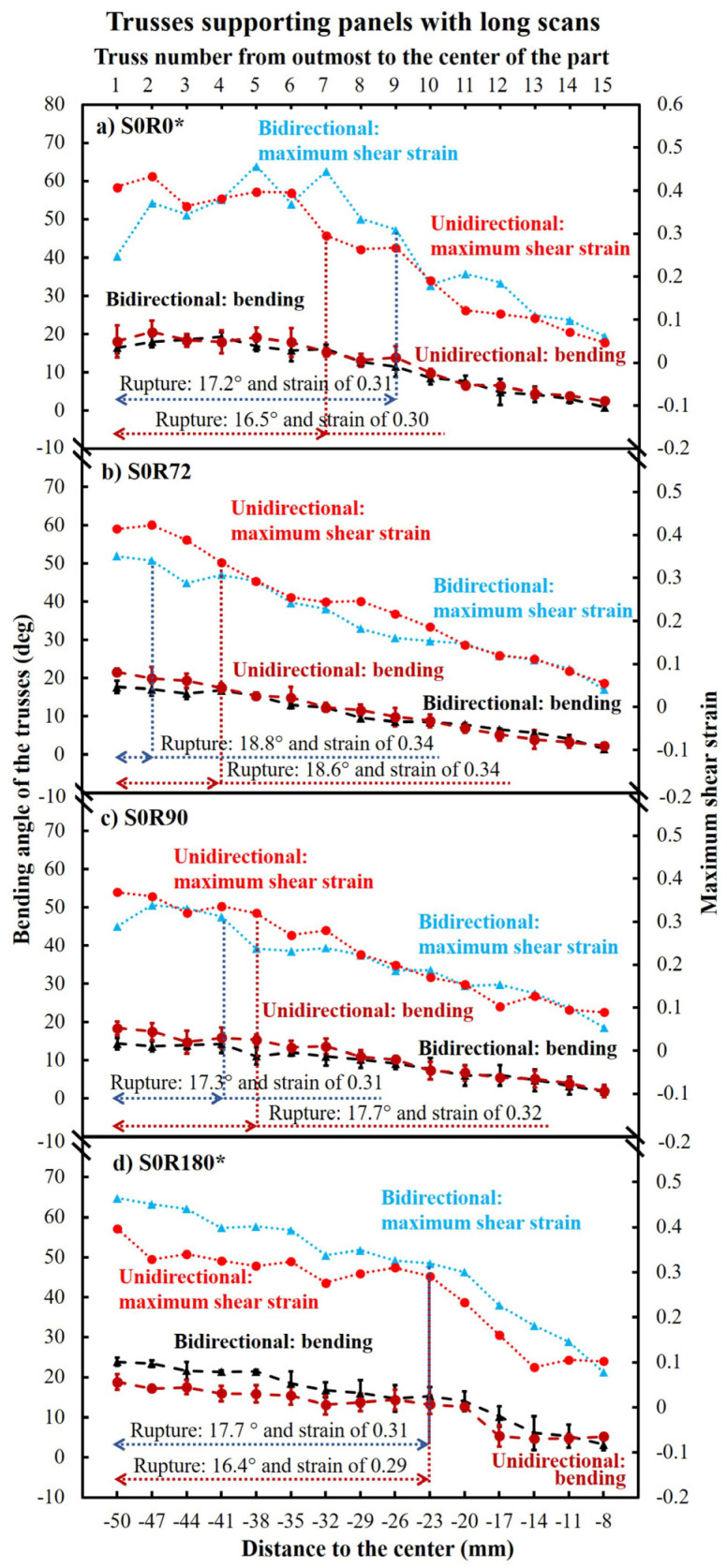
The experimentally measured bending angle and maximum shear strain (derived from maximum bending angle) of trusses supporting panels with long scans. These parts started with a 0° scan angle for their first layer and hence were subjected to a 100 mm long scanning length. * The unidirectional S0R0 and S0R180 panels were only 1.15 mm (instead of 2 mm) thick due to the process failure after excessive bending.

Figure 12 shows the measured bending angle and maximum shear strain (derived from the bending angle) imposed during EBM of the trusses underneath the panels made with long scans (see Table 2). As seen, the rotation angle influenced the rupture and bending. More specifically, the S0R0 (Figure 12a) and S0R180 (Figure 12d) parts had the highest number of ruptured trusses. The first trusses were not significantly different from those supporting S0R72 (Figure 12b) and S0R90 (Figure 12c) panels. Nevertheless, no meaningful correlation between the bending angle and failure can be observed from this figure. However, the maximum shear strain was the main factor controlling the fracture of the trusses, which turned out to be in the range of 0.29–0.34 and on average ~0.32 ± 0.02.

**Figure 13:**
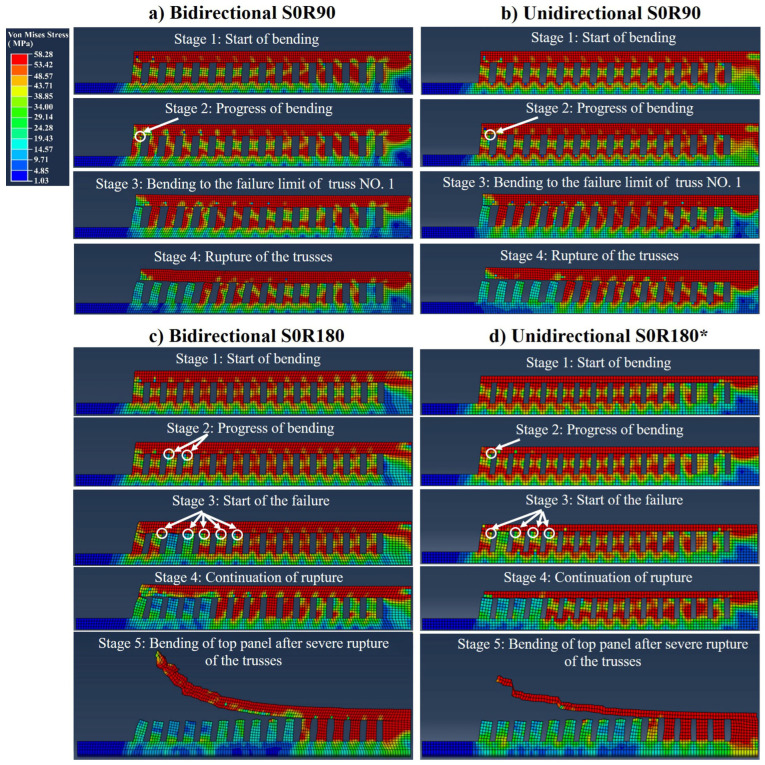
Stress simulated bending and rupturing of the trusses supporting (**a**) bidirectional and (**b**) unidirectional S0R90 panel as well as (**c**) bidirectional and (**d**) unidirectional S0R180 panel. Note that the unidirectional S0R180 part only had a panel thickness of 1.15 mm (instead of 2 mm) due to the process failure after excessive bending. For better visualization, see also the video provided in the supplementary data.

An ABAQUS simulation is performed for analysis of the bending data presented in Figure 12. Figure 13 demonstrates some examples from the simulated bending/rupture progress of S0R90 (Figure 13a,b) and S0R180 (Figure 13c,d) trusses made for panels with bidirectional and unidirectional scanning patterns. As seen, the bending/rupture progress is composed of different stages including bending start, the progress of bending, bending to the failure limit and initiation of rupture, and further rupture of the trusses. This was rather comparable for unidirectional and bidirectional scanned parts (e.g., see Figure 13a,b). However, the rotation angle had a more significant impact and the part with only long scans (e.g., S0R180) showed the highest number of ruptured trusses (see Figure 13c,d). After rupture, the failed trusses are stress relieved while the top panel may dramatically bend (see stage 5 in Figure 13c,d, as well as the video in supplementary data). Furthermore, it is interesting to observe that the stress did not always reach its maximum for the outmost truss for the panels with long scans (see S0R180 in Figure 13c,d). This could be due to the stress that develops first next to the outmost truss for this part.

**Figure 14:**
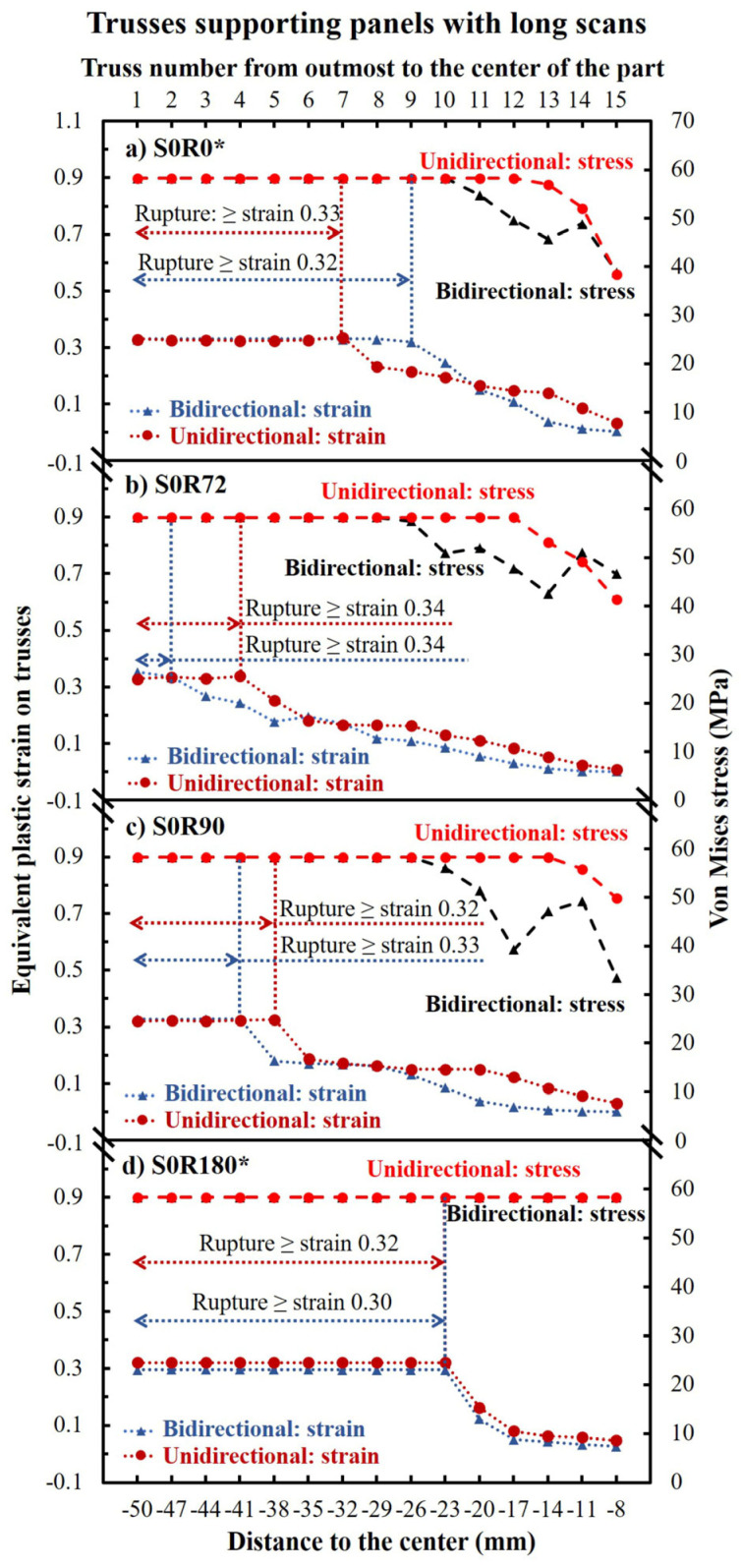
The equivalent plastic strain and von Mises stresses on each truss of the parts made with long scans. Note that the unidirectional S0R0 and S0R180 parts only had a panel thickness of 1.15 mm (instead of 2 mm) due to stopping the process after excessive bending. * The unidirectional S0R0 and S0R180 panels were only 1.15 mm (instead of 2 mm) thick due to the process failure after excessive bending.

From simulations, Figure 14 demonstrates the von Mises stresses and equivalent plastic strains on each truss of the parts made with long scans. As seen, all ruptured trusses reached a stress of 58 MPa and a strain of ~0.33 ± 0.01. While for the non-ruptured trusses, they can reach the UTS of material (58 MPa) but not the failure strain (<0.33) at 1050 °C. Moreover, it can be seen that the samples comprising both long and short scans (such as S0R72 and S0R90) may lead to comparable stresses. However, the associated effective plastic strains are less for such parts (see Figure 14 b,c). Lastly, the stresses and the strains at the parts made with a bidirectional scanning pattern were typically equal or slightly lower than that of those made with a unidirectional pattern.

#### 3.3.2. Short-Y Scanning Parts

**Figure 15:**
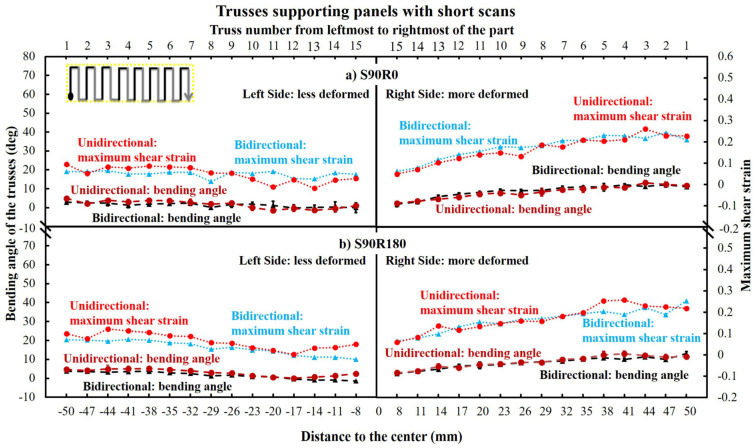
The bending angle and maximum shear strain (derived from maximum bending angle) for the supporting truss of the parts made with short-Y scans. Note that the bending of the trusses from two sides of the central supporting block are not symmetric.

Figure 15 demonstrates the bending and maximum shear strain (derived from the bending angle) of trusses underneath the panels built with short 10 mm scans and layer rotation angles of 0° and 180°. As seen, there is no significant difference imposed by rotation angles or bidirectional/unidirectional scanning patterns, which is different from the previous parts with longer scans. However, here the bending of the trusses was not symmetric, despite the bending being generally lower than the parts made with long scans (compare Figure 15 to Figure 12).

**Figure 16:**
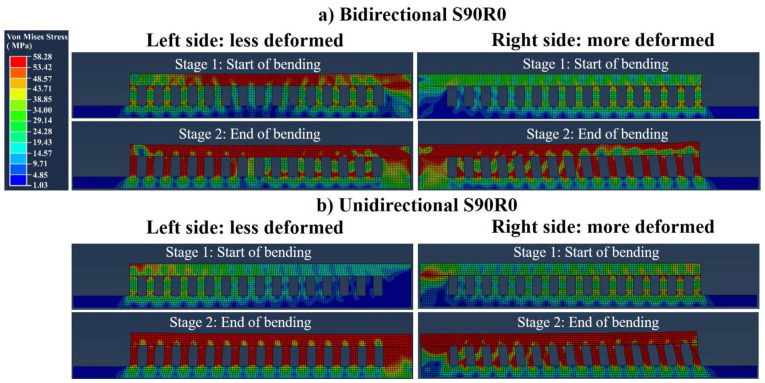
Stress simulated bending of the trusses for (**a**) bidirectional and (**b**) unidirectional S90R0 parts. Note that left side bent less compared to the right side of the parts.

Figure 16 shows the FE simulated stresses for the S90R0 part made with bidirectional and unidirectional scanning patterns. As seen, in both the parts made with bidirectional and unidirectional scanning, the bending is unsymmetrical since stresses do not develop exactly equally on the left and right sides of these specimens. This is due to the overall scanning direction at the first layer of the top panel which was from left to right, as schematically shown in Figure 15a (top left corner). This will be discussed further in Section 4.1. Additionally, since the imposed bending is small, no rupture occurs.

**Figure 17:**
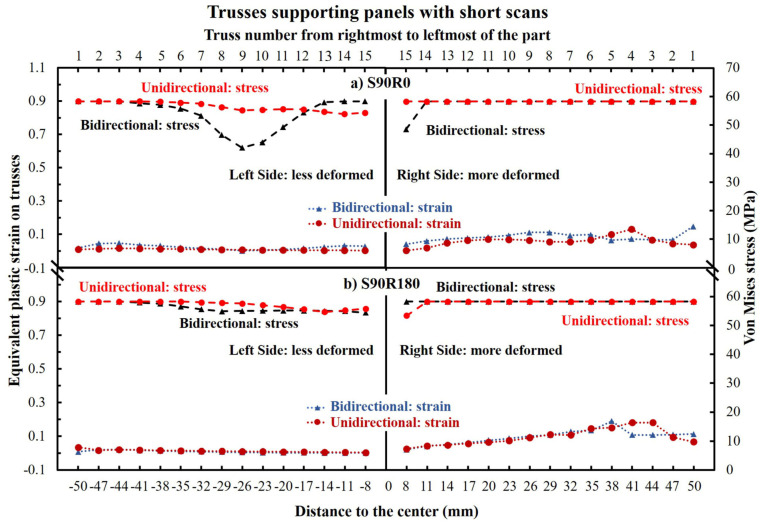
The equivalent plastic strain and von Mises stresses on each truss of the parts made with short-Y scans.

Figure 17 shows the simulated thermal stresses/stains of the trusses from short-Y scanning parts. As seen, the thermal stresses on each truss can remain high (including the trusses near the central supporting block). However, the strains of the trusses are below 0.2 which is much lower than long scanning parts. Accordingly, all the trusses were only bent but no rupture occurred. Moreover, even though the bending of the trusses from each side was different (Figure 15), the resultant thermal stresses were at an equal level, except bidirectional S90R0 which showed a minimum (a decrease until truss number 9 and then again an increase).

#### 3.3.3. Chessboard Scanning

**Figure 18:**
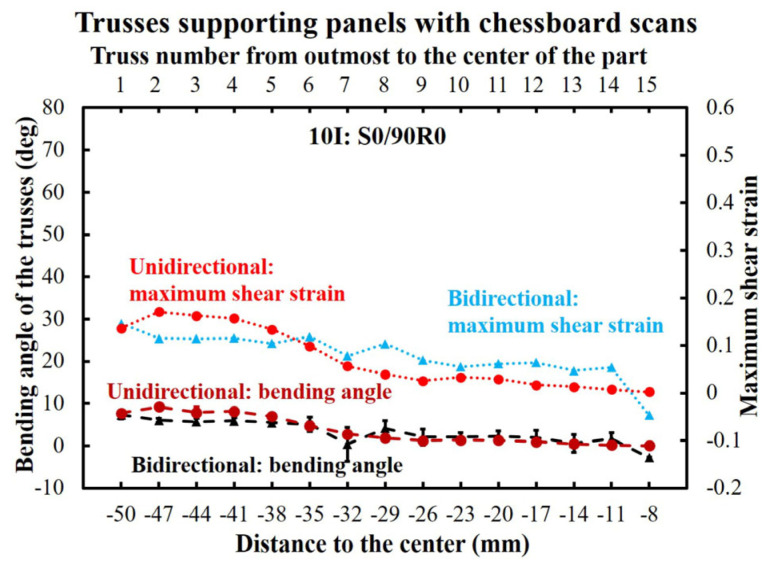
The bending angle and maximum shear strain (derived from maximum bending angle) of the chessboard printing.

Figure 18 plots the bending and maximum shear strain (derived from the bending angle) of the trusses for the chessboard scanned parts, which had shown no rupture after manufacturing. It is also observed that the bending had a smooth trend without showing any obvious difference between the bidirectional/unidirectional scanning patterns.

**Figure 19:**
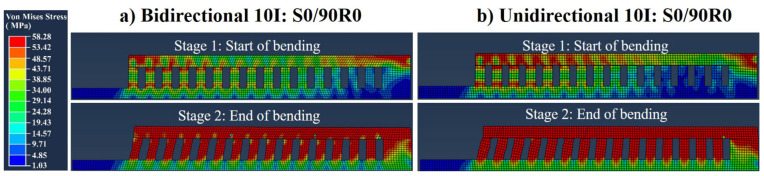
Simulated bending of the trusses for 10I:S0/90R0 parts: (**a**) bidirectional scanning pattern and (**b**) unidirectional scanning pattern.

In Figure 19, the simulated stresses of the bidirectional and unidirectional 10I:S0/90R0 parts are shown. The stresses were developed comparably between the bidirectional and unidirectional scanning, reaching 58 MPa for all the trusses despite no occurrence of failure.

**Figure 20:**
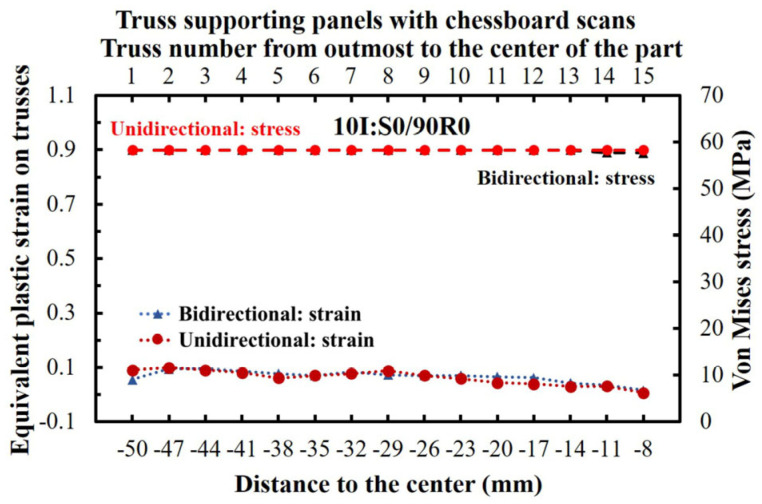
The equivalent plastic strain and von Mises stresses on each truss of chessboard scanned parts.

Figure 20 demonstrates the simulated von Mises stresses and equivalent plastic strains of the trusses from chessboard printed parts. The distribution of the thermal stresses was uniform, remaining constant at 58 MPa for all the trusses. This is while the equivalent plastic strain remained less than 0.1 (well-below the failure strain).

## 4. Discussion

### 4.1. Thermal Stresses and Developed Strains during EBM

During EBM, the trusses can bend differently according to the scanning length, the layer rotation, and the scanning path (unidirectional vs. bidirectional). The trusses supporting long scanning panels were always bent towards the center of the samples. This has occurred perhaps due to two collaborative reasons, related to the solid blocks in the middle of the panels. First, the solid blocks carry most of the heat transfer, inclining the shrinkage and consequently the bending of trusses inwards. Second, the solid blocks remain as stable anchors against unanchored outward directions, promoting easier inward shrinkage [15]. This is similar to the observations reported for SLM [9].

The part size is another factor, affecting the accumulative strains. In fact, the bigger the part, the larger the scanning. This increases the accumulative deformations and strains, particularly near the edges of the components [15,23,29]. Moreover, the long scans can lead to lower local temperatures and hence higher temperature gradients from the melt pool to the surroundings [44]. These higher temperature gradients increase thermal deformations [45]. As a result, it was observed that the trusses in S0R0 and S0R180 parts with only long scanning, had the most ruptures due to the accumulative strain, as well as their exposure to maximum temperature gradients. In comparison, 72° and 90° layer rotation (S0R72 and S0R90) utilized shorter scans in between the longer scans, and therefore, reduced the thermal deformations leading to ~56% fewer ruptured trusses. More successfully, short scans (i.e., S90R0, S90R180, and 10I:0/90R0) minimized the thermal deformation and consequently, no rupture occurred.

**Figure 21:**
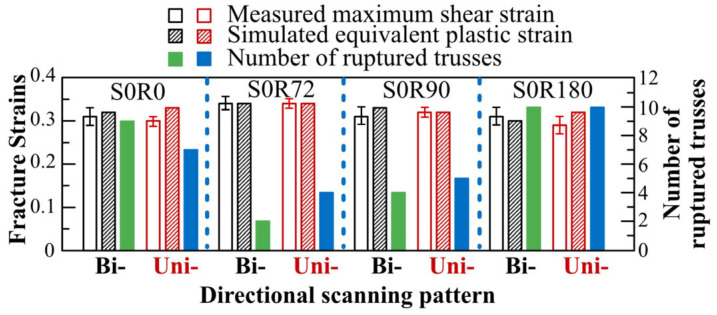
Comparison of fracture strains from experiments and simulations. For clarity, the total number of ruptured trusses have also been added for each case.

From the ruptured trusses, some interesting phenomena can be observed. To start, according to the FE simulations (Figure 14), all the ruptured trusses reached a von Mises stress of 58 MPa, which is equal to the UTS of the material at a high temperature. This also demonstrates that the stress of the remaining trusses can typically drop after the rupture of the trusses for the long scanning parts (i.e., stress relieves after rupture). In the case of no rupture, the stress can remain at nearly 58 MPa for almost all trusses (Figure 17 and Figure 20). Moreover, the rupture always occurred at a high strain of ~0.30–0.34 (Figure 14). The achieved equivalent plastic strain is very consistent with the maximum shear strain from experiments, as compared in Figure 21. In comparison, from the parts without ruptured trusses, the equivalent plastic strain never reaches more than 0.2. This is despite the fact that the thermal stress can reach 58 MPa UTS for these parts (see Figure 17 and Figure 20). These observations show that although a part can reach the UTS of the material at a high temperature during EBM, the rupture occurs only when the deformation/shrinkage is adequate to reach the maximum equivalent plastic strain (~0.33 ± 0.01 at this work). In other words, rupture during EBM is mainly strain-controlled, although their number could largely differ according to the applied scanning strategy.

**Figure 22:**
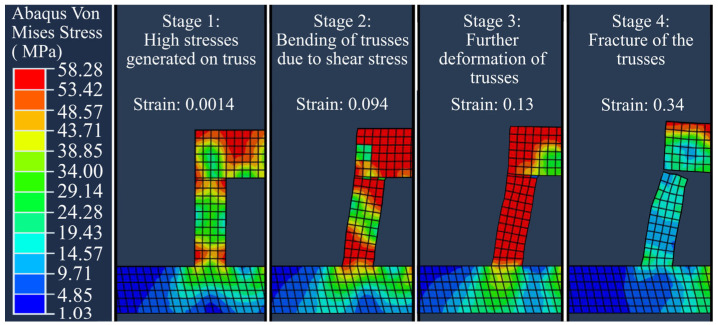
Typical bending progress of a truss until complete rupture via progress of von Mises stresses. The graph is taken from the unidirectional S0R72 part.

Figure 22 shows the typical bending progress until the rupture of a single truss, due to both tension and shear stresses. As seen, the stress grows until it reaches a maximum value of 58 MPa all over the truss, at an equivalent plastic strain level of about 0.13 (Figure 22 stage 1–3). Afterward, with further deformation the shear damage model takes effect, bending the truss. When the top reaches a strain of 0.34 at the integration points, the rupture initiates and hence the stress is relieved (Figure 22 stage 4). The truss bending behavior is similar to a thin cantilever beam [46] and consequently, large bending deformation is expected before rupture.

**Figure 23:**
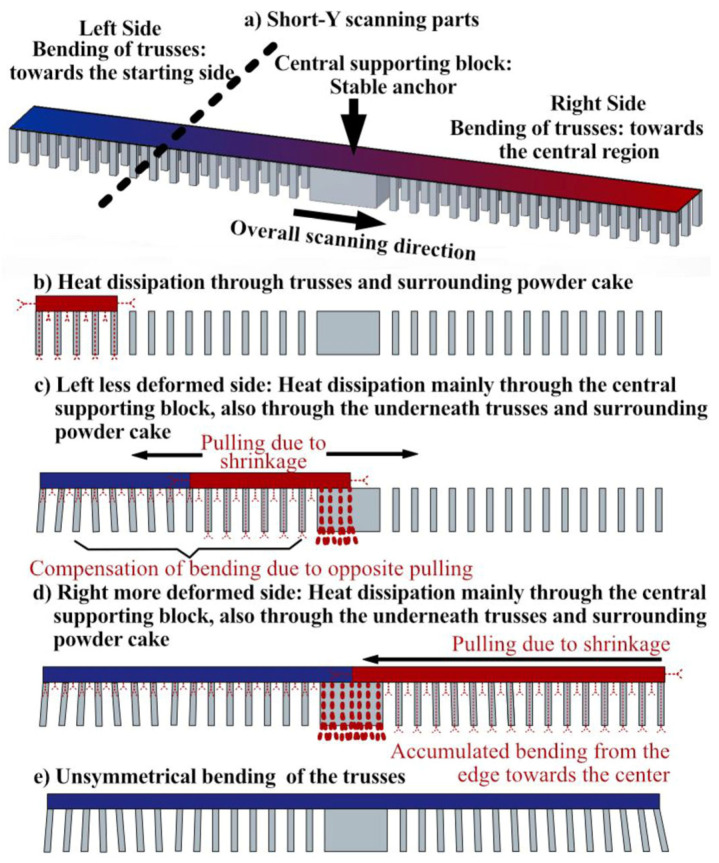
Schematic postulation for unsymmetrical bending of the trusses, supporting panels with short-Y scans.

As shown in Figure 15 to Figure 17, the trusses were bent non-symmetrically from the two sides of the central supporting block for the parts made with short-Y scans (S90R0 and S90R180). More specifically, the bending and the strain of the trusses on the right side were about twice as large as on the left side. This could be attributed to the reversing of the shrinkage direction at a threshold located somewhere around the middle of the left side, as schematically postulated in Figure 23.

As seen, while the beam moves along the part width, the first layer of the top panel melts and solidifies from left to right (Figure 23a). Naturally, the solidified region is pulled towards the heat dissipation, which is downwards and backwards at the start (Figure 23b). This inclines the trusses accordingly. However, after reaching a threshold, the direction of inclination seemingly changes possibly due to the heat conduction developed through the panel towards the central supporting block (Figure 23c). Afterward, the strains accumulate further, leading to more bending until the right edge (Figure 23d). This explains the unsymmetrical inclination of trusses (Figure 23e and Figure 15), which results in a peak value in the plotted stress (Figure 17).

**Figure 24:**
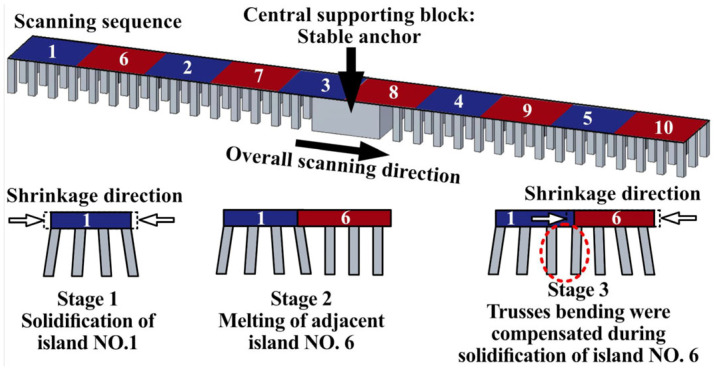
Schematic of the chessboard scanning strategy.

Unlike the short-Y scanned parts, the chessboard scanning led to a symmetrical bending since the same shrinkage was applied on both sides of the central supporting block. However, the overall bending for the chessboard scanning was observed to be somewhere between the left and right sides of the short-Y scanned parts (compare Figure 18 to Figure 15). This can be attributed to the fragmented scanning regions (i.e., melting one 10 mm × 10 mm island after another, as schematically shown in Figure 24). This means that the deflections can be compensated in various sequences from short 10 mm scans. This creates some irregularities in the bending of trusses (Figure 18), influencing the simulated strains as well (Figure 19).

### 4.2. Relation of Scanning Strategy to Part Quality in EBM

As described in the previous Section 4.1, longer scans increased the bending and ruptures, despite the thermal stresses being typically at a maximum value for most of the trusses. Consequently, the layer rotation angle reduced the accumulative bending and ruptures (Figure 14a–c), since the layer rotation angle together with the starting scanning angle decreased the scanning lengths. Additionally, rotation between layers allowed better consolidation between layers, and therefore, a higher density (Figure 10). Accordingly, a certain rotation between layers is always recommended which can typically lead to a simultaneous reduction in porosity and thermal deformation [20,47].

Ultimately, when only short scans were employed even without any rotation (S90R0, S90R180), the density can be maximized. This can be attributed to the higher local temperatures from shorter scans, since the beam returns to its previous adjacent location faster [9], in agreement with other findings reported by Everhart et al. for EBM of Ti_6_Al_4_V [44]. Additionally, the rupture could be fully avoided for short scans, as only a little strain developed for these parts. This is similar to the SLM process [20,21], where the short scans are favored in order to reduce the thermal deformations/strains of the trusses. This can be related to opposite shrinkage during the scanning of this sample, which interrupted the accumulation of strains. The only issue with such conditions comes from the fact that bending of the trusses may end unsymmetrical. The unsymmetrical bending (Figure 23) can further result in geometrical inaccuracies. However, this is rationally rather negligible compared to the fact that there is no excessive bending and no truss rupture; hence no serious warpage is expected.

Regarding the chessboard strategy, even though the chessboard scanning minimized the bending and did not allow any rupture of the trusses (comparable to the short-Y scanned parts), cracks could form at the overlapping region between the adjacent islands (Figure 11). This is due to the specific melting sequence, as the islands were melted one after another, the cooling of the adjacent islands could be in different directions (Figure 24). Therefore, the overlapping regions can shrink in different directions creating stresses and large cracks between adjacent islands. Thus, the parts made with chessboard printing could not reach as a high density as the parts made with the default setting (S0R72) and short-Y scans (S90R0 and S90R180) [21], as shown in Figure 10.

In addition to the scanning length, the bidirectional vs. unidirectional scanning pattern is another important parameter influencing the generated thermal stresses/strains. After analyzing the influence of the bidirectional or unidirectional scanning patterns on the development of thermal stresses and strains (Figure 14, Figure 17 and Figure 20), it seems that bidirectional scanning may typically create a slightly lower equivalent plastic strain despite comparable stress levels. It should be noted that this general trend is clearer for the long scans (Figure 12, Figure 13 and Figure 14), although it is rather negligible for the short ones. This could be related to no jumping of the beam and hence no interval between scanning lines in bidirectional scanning. This may slightly increase the overall part temperature (which is in line with the slightly higher density and part adhesion for the bidirectional pattern, see Figure 10). Therefore, this reduces the temperature gradient, decreasing the part deformation [48]. It is also interesting to note that particularly for the bidirectional scanning, which increases the temperature and shrinkage at the ending points, the rupturing of the trusses may not initiate from the outmost truss for the panels with long scans (see S0R180 in Figure 13c,d). This is because the outmost truss had a free surface that carries no shear with zero principal stress [49]. The stress and strain were then developed with the distortion of the top panels. Thus, it can later rupture after the ruptures of nearby trusses.

According to the above discussions, one can conclude that the application of short scans leads to the smallest thermal strains and deformations in addition to increasing the density. However, to implement short scans, chessboard scanning might not be the best option since poor chessboard parameters may promote hot tears and cracks at the overlapping regions of the neighboring islands. Furthermore, bidirectional scanning was found to be the safest scanning pattern, slightly improving the density and reducing the imposed strains. Accordingly, to minimize part failure in EBM, it is suggested to rotate the scanning direction with respect to the longer length of the parts to avoid long scans while using a bidirectional scanning pattern.

## 5. Conclusions

This work analyzed the effect of scanning length, uni/bi-directional patterns, layer rotation, and chessboard scanning on the generation of thermal stresses/strains, as one of the top sources of the build failure during EBM of IN625. The results have shown that:

- The thermal stresses on the trusses from different scanning strategies reach the UTS of IN625 (58 MPa) during EBM (1050 °C in this work). However, this does not mean that the part necessarily fails. In fact, failure is rather strain-controlled since it only occurs when the plastic strain increases to a certain level (around 0.33 in this work, according to both experiments and simulations);
- The scanning length was the main parameter to control the induced thermal plastic strain. Shorter scanning lines were always favored since they prevented the accumulation of strains in long lengths. In addition, shorter scans had a slightly positive influence on density due to increasing the local temperature. However, it is noted that the short-Y scanning strategy led to an unsymmetrical bending between the two sides of the deflection sample which has not been reported as an issue for the SLM process. This was attributed to the change in heat transfer and hence shrinkage direction during EBM;
- The rotation angle in each layer was another important parameter that determined the scanning length. Accordingly, since a proper rotation angle decreased the scanning length in successive layers, a layer rotation was recommended to reduce the thermal deformation;
- Interestingly, unlike the SLM process, the differences in thermal stresses/strains between bidirectional and unidirectional patterns were rather small and even negligible in some cases.
- The chessboard scanning can effectively mitigate the bending and prevent the rupture of the trusses. This was due to the short scanning lengths of the islands. However, large cracks could form at the overlapping scanning regions due to the opposite pulling of the adjacent islands;
- In summary, to minimize part failure in EBM due to thermal deformations, it is suggested to use a bidirectional scanning pattern while rotating/orienting the scanning direction in such a manner that one can deliver the part using shorter scans. This can be a lesson for machine software developers;
- Moreover, as recognized from this work, the processing temperature for IN625 using EBM should be lower. A proper processing temperature should be below the embrittlement temperature for such an alloy.

## Figures and Tables

**Table 1:** The selected scenarios for the thermal stress analysis.

Scanning	Sample ID	NO. of Islands	Starting Angle	Rotation Angle per Layer (°)
Long	S0R0	1	0	0
	S0R72	1	0	~72 (Default setting)
	S0R90	1	0	90
	S0R180	1	0	180
Short-Y	S90R0	1	90	0
	S90R180	1	90	180
Short Chessboard	10I:S0/90R0	10	0/90	0

**Table 2:** The summary of different parts according to their different scanning angle, length and time.

Scanning	Sample ID	Layer Number	Scanning Angle (°)	Average Scanning Length (mm)	Layer Scanning Duration (s)	
					Bidirectional	Unidirectional
Long	S0R0	every	0	100	1.99 ± 0.28	2.07 ± 0.34
	S0R72	n	0	100	1.99 ± 0.28	2.07 ± 0.34
		n + 1	72	10.51	13.84 ± 2.50	15.46 ± 2.97
		n + 2	144	17.01	4.40 ± 0.16	4.79 ± 0.20
		n + 3	216	17.01	6.18 ± 1.51	6.67 ± 1.61
		n + 4	288	10.51	13.10 ± 1.38	14.22 ± 1.74
	S0R90	n	0	100	1.99 ± 0.28	2.07 ± 0.34
		n + 1	90	10	14.20 ± 1.89	15.76 ± 2.25
		n + 2	180	100	1.72 ± 0.27	2.13 ± 0.20
		n + 3	270	10	14.19 ± 1.83	15.66 ± 2.15
	S0R180	n	0	100	1.99 ± 0.28	2.07 ± 0.34
		n + 1	180	100	1.72 ± 0.27	2.13 ± 0.20
Short-Y	S90R0	every	90	10	14.20 ± 1.89	15.76 ± 2.25
	S90R180	n	90	10	14.20 ± 1.89	15.76 ± 2.25
		n + 1	270	10	14.19 ± 1.83	15.66 ± 2.15
Short chessboard	10I: S0/90R0 *	every	0	10	7.70 ± 1.02	8.35 ± 1.17
			90	10	7.88 ± 1.08	8.90 ± 1.27

* The scanning times are varied based on the area of melting region. For the 10I:S0/90R0, the part comprises of five islands with 0° scanning angle and five islands with 90°. This can impose different scanning times.

**Table 3:** Material information for IN625 used for ABAQUS simulation.

Density (g/cm^3^)	Young’s Modulus (GPa) *	Poisson’s Ratio	Yield Stress and Plastic Strain *	
			Stress (MPa)	Plastic Strain
8.44	45	0.33	46	0
			55.4	0.00035
			58	0.0015

* These values were obtained from the high temperature tensile testing.

**Table 4:** The summary of the manufactured size of the supporting trusses.

Scanning	Sample ID	Bidirectional			Unidirectional		
		Width (mm)	Thickness (mm)	Height (mm)	Width (mm)	Thickness (mm)	Height (mm)
Long	S0R0	1.7 ± 0.14	1.0 ± 0.10	3.7 ± 0.04	1.5 ± 0.09	1.0 ± 0.08	3.7 ± 0.09
	S0R72	1.3 ± 0.03	1.1 ± 0.06	3.8 ± 0.06	1.5 ± 0.05	1.3 ± 0.05	3.8 ± 0.03
	S0R90	1.6 ± 0.04	1.2 ± 0.04	3.7 ± 0.12	1.7 ± 0.09	1.3 ± 0.10	3.7 ± 0.05
	S0R180	1.8 ± 0.07	1.1 ± 0.01	3.6 ± 0.01	2.0 ± 0.07	1.2 ± 0.03	3.8 ± 0.05
Short-Y	S90R0	1.2 ± 0.02	1.2 ± 0.04	3.8 ± 0.06	1.3 ± 0.10	1.3 ± 0.03	3.7 ± 0.06
	S90R180	1.2 ± 0.04	1.2 ± 0.04	3.8 ± 0.07	1.3 ± 0.04	1.3 ± 0.03	3.8 ± 0.06
Short chessboard	10I: S0/90R0	1.6 ± 0.08	1.5 ± 0.07	3.6 ± 0.05	1.7 ± 0.07	1.7 ± 0.03	3.7 ± 0.08
CAD model	Designed	1	1	5	1	1	5

## Data Availability

Not applicable.

## Supplementary Materials

The following supporting information can be downloaded at: https://www.mdpi.com/article/10.3390/ma16010443/s1.
